# A 4-gene signature from histologically normal surgical margins predicts local recurrence in patients with oral carcinoma: clinical validation

**DOI:** 10.1038/s41598-020-58688-y

**Published:** 2020-02-03

**Authors:** Patricia P. Reis, Tomas Tokar, Rashmi S. Goswami, Yali Xuan, Mahadeo Sukhai, Ana Laura Seneda, Luis E. S. Móz, Bayardo Perez-Ordonez, Colleen Simpson, David Goldstein, Dale Brown, Ralph Gilbert, Patrick Gullane, Jonathan Irish, Igor Jurisica, Suzanne Kamel-Reid

**Affiliations:** 1 São Paulo State University, UNESP, Faculty of Medicine, Department of Surgery and Orthopedics, Botucatu, SP Brazil; 2 Krembil Research Institute, University Health Network, Toronto, ON Canada; 3 Department of Clinical Pathology, Sunnybrook Health Sciences Centre, Toronto, ON Canada; 4 Princess Margaret Cancer Centre, University Health Network, Toronto, ON Canada; 5 Brazilian Institute for Cancer Control, São Paulo, SP Brazil; 6 Department of Pathology, Toronto General Hospital, University Health Network, Toronto, ON Canada; 7 Departments of Medical Biophysics, University of Toronto, Toronto, ON Canada; 8 Department of Computer Science, University of Toronto, Toronto, ON Canada; 9 Institute of Neuroimmunology, Slovak Academy of Sciences, Bratislava, Slovakia; 10 Clinical Laboratory Genetics, Genome Diagnostics, University Health Network, Toronto, ON Canada; 11 Department of Laboratory Medicine and Pathobiology, The University of Toronto, Toronto, ON Canada

**Keywords:** Prognostic markers, Oral cancer

## Abstract

Prognostic biomarkers for recurrence of Oral Squamous Cell Carcinoma (OSCC) are urgently needed. We aimed to independently validate a 4-gene expression signature (*MMP1*, *COL4A1*, *P4HA2*, *THBS2*) predictive of OSCC recurrence risk. Gene expression was measured using Nanostring nCounter^®^ in 245 histologically normal surgical resection margins from 62 patients. Association between risk scores for individual patients and recurrence was assessed by Kaplan-Meier analysis. Signature performance was quantified by concordance index (CI), hazard ratio (HR) and the area under receiver operating characteristics (AUC). Risk scores for recurrence were significantly higher than recurrence-free patients (*p* = *9*.*58e-7*, Welch’s t-test). A solid performance of the 4-gene signature was determined: CI = 0.64, HR = 3.38 (*p* = *1*.*4E-4*; log-rank test), AUC = 0.71. We showed that three margins per patient are sufficient to preserve predictive performance (CI = 0.65; HR = 2.92; *p* = *2*.*94e-3*; AUC = 0.71). Association between the predicted risk scores and recurrence was assessed and showed HR = 2.44 (*p* = *9*.*6E-3*; log-rank test, N = 62). Signature performance analysis was repeated using an optimized threshold (70^th^ percentile of risks), resulting in HR = 3.38 (*p* = *1*.*4E-4*; log-rank test, N = 62). The 4-gene signature was validated as predictive of recurrence risk in an independent cohort of patients with resected OSCC and histologically negative margins, and is potentially applicable for clinical decision making on adjuvant treatment and disease monitoring.

## Introduction

Oral Squamous Cell Carcinoma (OSCC) is the most common head and neck cancer, and the 8^th^ most frequent cancer^1^, accounting for >300,000 new cases and >145,000 deaths every year, worldwide^2^. Conventional treatment for OSCC includes surgery, radiotherapy and chemotherapy. However, patients often exhibit poor response to treatment and high rates of recurrence, typically between 20–40%^3^, reducing the five-year survival to 50%^4^.

One important factor associated with improved patient survival is complete surgical removal of the primary tumor^3^. Presence of epithelial dysplasia or tumor cells in the surgical resection margins is known to be associated with a higher risk of disease recurrence^5^. However, even with histologically normal surgical resection margins, a 10–30% recurrence rate is still observed^6^. Therefore, histologically normal margins may harbor molecular changes, leading to their malignant transformation and disease recurrence^7,8^. Previous studies identifying molecular changes in surgical margins in head and neck cancer, including OSCC, are outlined in Supplementary Table 1, with corresponding literature citations. Most of the previous work used a candidate gene or protein approach instead of global profiling, and were based on the analysis of distinct cancer sites in the head and neck, which have different biological and clinical behaviors. More importantly, there is a lack of comprehensive validation using independent samples.

In our previous work^9^, we identified a 4-gene signature (*MMP1*, *COL4A1*, *P4HA2* and *THBS2*) predictive of OSCC local recurrence in a set of 96 margins from 24 patients, and validated our findings in an independent set of 136 histologically normal surgical margins from 30 OSCC patients. Here, we provide extended, independent, retrospective validation of this signature on a new, independent cohort of 245 histologically normal surgical margins from 62 patients with OSCC, using the NanoString nCounter assay^10^. This analysis confirmed that the 4-gene signature accurately predicts recurrence risk in patients with OSCC and histologically normal (negative) surgical resection margins.

## Results

### Patient characteristics

Out of 62 patients, 34 (55%) had local recurrence, with median time to recurrence of 10.5 months after surgery (Table 1). There was a statistically significant association between recurrence and age (CI = 0.57, HR = 2.35, p = 1.36e-2, log-rank test, AUC = 0.62), but not gender, smoking status, alcohol consumption, tumor stage, grade, or tumor subsite (Table 2). Interestingly, no significant association between recurrence and age or other clinical characteristics was found in our previous study^9^.

**Table 1:** Clinicopathological characteristics of patients in this study and in Reis *et al*.^9^.

Characteristics		Value		
		Current study (independent validation)	Reis *et al*. 2011 (training)	Reis *et al*. 2011 (validation)
		N = 62	N = 24	N = 30
Sex:	Female	27	9	9
	Male	35	15	21
Age [years]:	Median	64.5	58.5	67
	Mean	62.7	59.4	65.2
	Range	27–83	37–83	48–85
Tumor stage:	I	11	1	4
	II	17	7	9
	III	15	3	7
	IV	15	13	10
Tumor grade:	Well	17	0	0
	Moderate	41	18	23
	Poor	2	6	7
Recurrence:	No	28	15	23
	Yes	34	9	7
Time to recurrence [months]:	Median	10.5	3.2	8
	Mean	17.6	12.2	15.1
	Range	2.9–86.3	1.8–34	2–36
Follow-up [months]:	Median	63	13	21
	Mean	75	24.8	26.7
	Range	5–199	1.7–58.8	1–81
Smoking:	Never	23	8	5
	Former	15	16	25
	Current	23	0	0
Alcohol consumption:	No	20	7	9
	Yes	40	17	21

**Table 2:** Prognostic ability of clinical characteristics.

Characteristics	HR	*p*-value	*N*	*n*
Age	2.35	0.0136	62	2
Sex	0.86	0.6517	62	2
Tumor stage	1.26	0.7880	58	4
Tumor grade	2.29	0.1561	60	3
Smoking status	1.36	0.0732	61	3
Alcohol consumption	0.61	0.1632	60	2
Tumor subsite	1.22	0.5704	62	2

*N* denotes number of patients used and *n* indicates number of groups, patients were stratified into, to assess the given characteristics.

In case of age assessment patients were stratified using median age (64.5) as a threshold.

### Validation of the signature

**Figure 1:**
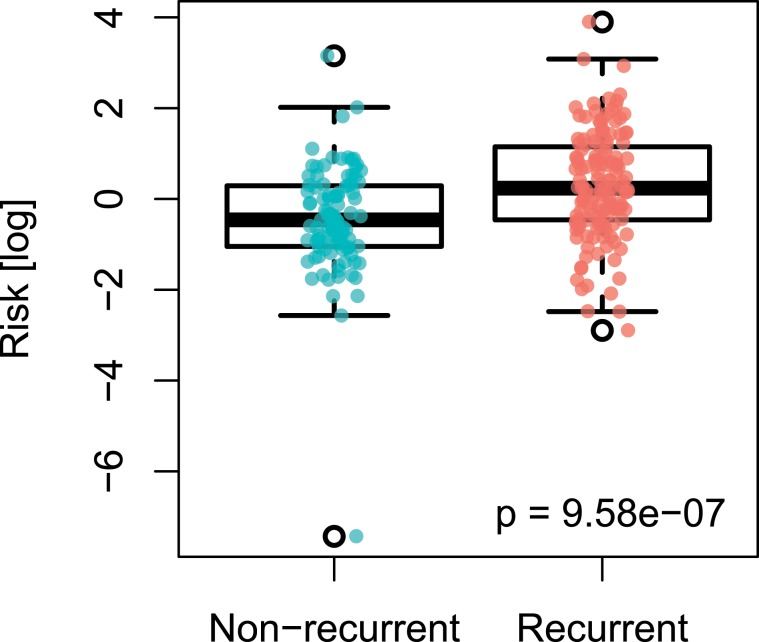
Boxplot depicting distribution of risks derived from margins samples of patients without (n = 28; 45%) and with locally recurrent tumors (n = 34; 55%).

For each of the margin samples we first calculated sample-specific risks, derived from signature gene expression values as obtained from individual samples (Supplementary Fig. 1). We compared the risks derived from the samples of patients with recurrence to those from patients without recurrence. We found that risks derived from recurrence-positive samples (n = 34; 55%) were significantly higher than the risk score from recurrence-negative samples (p = 9.58e-7, Welch’s t-test; Fig. 1).

**Figure 2:**
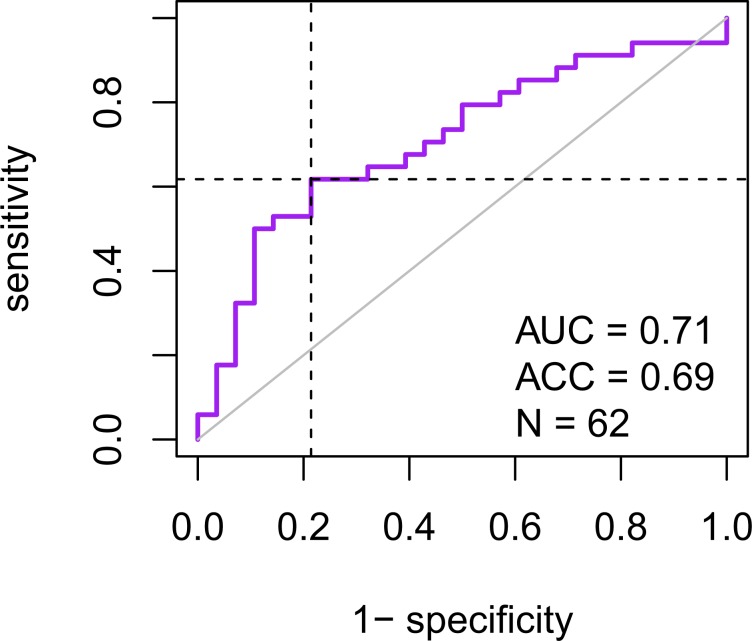
Receiver operating characteristics depicting patient classification performance of the signature. Horizontal and vertical lines indicate sensitivity and false positive rate (1 – specificity) achieved when using median risk as the threshold. Associated statistics, including area under the curve and accuracy, are listed in the bottom right corner.

**Figure 3:**
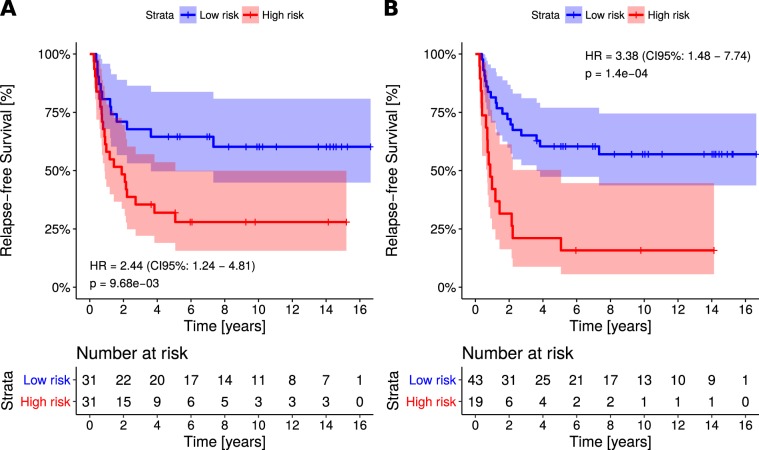
Kaplan-Meier plot depicting survival of the patients with low risk (LR) and high risk (HR) as estimated by applying the signature, stratifying patients using the median of the obtained risks (**A**) and optimized threshold, equal to 70^th^ percentile of the obtained risks. (**B**) Colour-shaded areas depict 95% confidence intervals of the survival curves. Obtained statistics are listed in the bottom left corner.

For each patient, we then predicted the risk of local recurrence using the maximum gene expression value across all margins. The 4-gene signature was first evaluated by concordance index (CI), between predicted risk and recurrence-free survival time. The resulting value (CI = 0.64) indicated a solid predictive performance of the signature. The area under the receiver operating characteristics curve (AUC) was calculated, resulting in AUC = 0.71, further highlighting the predictive performance of the signature (Fig. 2). Calculating Spearman rank correlation between predicted risks and age at diagnosis showed ρ = −0.047 (p = 0.71, randomization test), excluding possible confounding effect of age. Patients were then stratified into two groups using median risk as a threshold. Association of the predicted risk and recurrence was assessed by calculating hazard ratio (HR). The resulting values: HR = 2.44 (p = 9.6E-3; log-rank test, N = 62; Fig. 3a) validated our published results^9^. This was then repeated using an optimized threshold (70^th^ percentile of risks), resulting in HR = 3.38 (p = 1.4E-4; log-rank test, N = 62; Fig. 3b).

**Figure 4:**
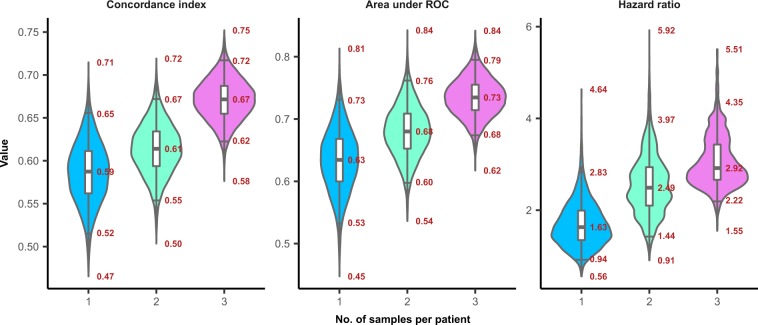
Violin plots depicting distributions of the major indicators of the signature performance, including concordance index (CI), area under ROC (AUC) and hazard ratio (HR), obtained using one, two and three randomly chosen samples per patient to assess patient risk. Annotations show minimum, 2.5^th^ percentile, median, 97.5^th^ percentile and maximum of the given distributions.

To assess how the reduced number of margin samples affects the 4-gene signature performance, we predicted the recurrence risk for each patient using gene expression from only one, randomly selected margin sample. CI, AUC and HR (using 70^th^ percentile of risks as a threshold) with their respective p-values, were subsequently determined to evaluate the predictive performance. This was repeated 10,000 times, resulting in median values of HR = 1.63 (p-value = 0.16, log-rank test, N = 62), CI = 0.59, AUC = 0.63 (Fig. 4). Resulting values demonstrate substantial decline of the signature performance. This further corroborates our previous results^9^, leading to the conclusion that expression changes of this 4-gene signature are spread heterogeneously across the margins, and the use of only one or a reduced number of margins for molecular analysis decreases the probability of detecting gene expression changes associated with recurrence.

To further investigate how many margins are needed to provide robust recurrence prediction, the same Monte-Carlo analysis was applied herein, using maximum expression of genes, as calculated across two and three randomly selected margins per patient. Patients with insufficient number of margins samples were omitted. Using two different margins samples per patient resulted in median values of: HR = 2.49 (p-value = 8.1e-3, log-rank test, N = 57), CI = 0.61, AUC = 0.68 (Fig. 4). Using three different margin samples per patient results in median values of: HR = 2.92 (p-value = 2.94e-3, log-rank test, N = 47), CI = 0.67, AUC = 0.73 (Fig. 4), comparable to the values observed when using a full set of samples across the whole cohort of patients.

### Functional assessment of the signature genes

To provide functional analysis of the signature genes we further performed a comprehensive pathway enrichment analysis using validated head and neck tissue-specific PPI partners. We identified 43 significantly enriched pathways (FDR < 0.01; Supplementary Fig. 2). The most enriched pathways include organization of extracellular matrix, cell surface interactions of Beta1 and Beta3 integrins, collagen synthesis, focal adhesion, PI3K-Akt signalling, and miRNA targets in extracellular matrix and membrane receptors.

## Discussion and Conclusions

Prognostic models require independent validation studies before can be considered as clinically useful^11^. Therefore, there is a need to demonstrate an ability to distinguish patients with favorable from poor outcome across multiple independent cohorts, and with an alternative analysis method. In order to satisfy these requirements, we performed an extended validation of the previously developed 4-gene prognostic signature, using an independent cohort of patients as well as an alternative, probe-based assay (Nanostring nCounter^®^) for obtaining gene expression data. The limitation of this study is the lack of participants from geographically distinct populations.

Considering CI, HR and AUC and a full set of patient samples we have validated results obtained by the original study^9^. Current results demonstrate the robust predictive performance of the 4-gene signature, and thus verify our previous results using an independent patient cohort and a new gene expression assay platform. In addition, we showed that the prediction accuracy depends on the number of separate margin samples across which the expression data are integrated.

Since genetic aberrations may spread from the tumor asymmetrically, they may be undetectable in some of the obtained margins^9^. A greater number of available margin samples increases the chance of detection of these aberrations and enables a more precise quantification of patient risk. Therefore, the inclusion of multiple histologically negative margin samples per patient for gene expression analysis is one of the strengths of our study. Our results show that the current signature demonstrates sufficient prediction accuracy if at least three separate margins per patient are used. This requirement is clinically feasible and should not prevent utilization of the signature in clinical practice.

Pathway enrichment analysis using the signature genes and their PPI partners revealed their involvement in several pathways known to be associated with OSCC development. These include organization of the extracellular matrix, integrin interactions and collagen synthesis; changes of which are essential for many malignant processes in OSCC^12^. Similarly, PI3K-Akt signalling alterations are observed in multiple cancers, and are also believed to be associated with cancer drug resistance^13^. A recently identified partial-epithelial to mesenchymal transition (p-EMT) program has been shown as an independent predictor of nodal metastasis, tumor grade and additional pathological features indicating aggressive tumor behaviour in oral carcinoma^14^. Notably, our signature genes are among the study findings, with *MMP1* being over-expressed in p-EMT cells, and *COL4A1*, *P4HA2* and *THBS2* up-regulated upon TGFb treatment, which induced a p-EMT-like program and increased invasive potential, in the SCC9 oral cancer cell line. This study corroborates our findings, and we highlight the existence of molecular alterations in surrounding tumor tissues, including surgical resection margins, as drivers of disease recurrence.

Our findings have translational relevance for the management of patients with OSCC. Patients with a diagnosis of histologically negative margins are often considered to have a lower risk of recurrence; however, we demonstrate that a subset of these patients develops recurrent disease and that recurrence can be predicted by gene expression changes. In the absence of other clinical indicators, prediction of a high risk of local recurrence in patients with histologically negative margins could thus be used to modify post-operative treatment options and surveillance. We suggest clinical utilization of this 4-gene signature for molecular analysis of histologically negative margins from patients undergoing OSCC surgical resection as part of post resection clinical management.

## Material and Methods

This is a retrospective, single center cohort study aimed to independently validate the 4-gene prognostic signature. This study complies with the Guidelines for the REporting of tumor MARKer Studies (REMARK)^15^, and followed the guidelines outlined in the Transparent Reporting of a multivariant prediction model for Individual Prognosis Or Diagnosis (TRIPOD) statement^16^. Original, raw data were submitted to Gene Expression Omnibus (GEO), and are publicly available under GEO series number **GSE108712**.

### Ethics declaration – study participants

This work was performed under approval of the University Health Network Research Ethics Board (REB approval number 02-0488-C). The study followed the ethics recommendations from the Helsinki Declaration. Informed consent was obtained from all patients before sample collection. Patients included in the study were diagnosed and treated at the Princess Margaret Cancer Centre/Toronto General Hospital, Toronto, ON, Canada. Patient inclusion criteria were: (i) primary, surgically resected OSCC, (ii) patients having a final pathology report confirming that all surgical resection margins were histologically normal or negative.

### Clinical specimens and study design

#### Sample size

We retrospectively collected formalin-fixed, paraffin embedded (FFPE) tissue blocks from 277 margin samples from 67 patients. Of these, 8 samples were removed since they were technical replicates. Additional 24 samples from five patients with insufficient follow up time (follow up <3 years). This study was conducted on 245 margins samples obtained from a total of 62 patients, each with more than 3 years of follow-up. We have analyzed up to 8 surgical resection margins per patient, with an average of 4 margins per patient. All margins were histologically normal (i.e., negative), as determined by final histopathological analysis (BP-O). None of the patients received chemo or radiation therapy before surgery and sample collection.

#### Source of data

Sample accrual and retrieval was performed starting in March, 2011 and ending in March, 2012. RNA was extracted in April-May, 2012. Gene expression analysis was performed in June, 2012. Samples were randomized and their identities anonymized using the Random Sampling feature in Microsoft Excel prior to gene expression analysis.

#### Outcome measures

Patients were classified into two groups: (1) patients experiencing local recurrence and (2) recurrence-free patients. Recurrence status was updated in September, 2019 (C.S., clinical research assistant). Pathology reports were examined to confirm recurrence status, which is established by the head and neck oncology team based on standard examination procedures for patients with OSCC (PG, JI).

#### Procedures

FFPE tissues from histologically normal surgical resection margins were cut (4–5 µm tick), stained with Hematoxylin-Eosin and subjected to needle microdissection using the stereo microscope Leica EZ4 (Leica Microsystems, Wetzlar, Germany) before RNA extraction, in order to isolate pure normal cell populations from margin tissues. RNA from FFPE samples was isolated using the RecoverAll Total Nucleic Acid Isolation kit (Ambion/Life Technologies, Carlsbad, CA, USA), following our previously reported protocol with modifications to improve RNA yield^17^. RNA was stored at −80 °C until RNA extraction and gene expression validation. In addition, tumor tissues from the same patients were collected to confirm whether the 4-gene signature was also deregulated in tumors, in the same direction (up-regulation) as compared to surgical resection margins.

Two Nanostring nCounter^TM^ probes were designed against each gene of the 4-gene signature (*MMP1*, *COL4A1*, *P4HA2* and *THBS2*), being comprised of one capture probe linked to biotin and one reporter probe attached to a color-coded molecular tag for each gene sequence, according to the nCounter^TM^ code-set design. RNA samples randomized by a numerical ID were subjected to NanoString nCounter^TM^ analysis by the Princess Margaret Genomics Centre (https://www.pmgenomics.ca) facility, Toronto, ON, Canada.

Patient samples were analyzed in two batches consisting of 125 and 152 margins tissue samples. The detailed protocol for mRNA transcript quantification analysis, including sample preparation, hybridization, detection and scanning followed the manufacturer’s recommendations and was described in^18^. We used 400 ng of total RNA isolated from FFPE tissues for detection of probe signals. Raw data were analyzed using the nCounter^TM^ digital analyzer software (http://www.nanostring.com/support/ncounter), and gene expression levels were subjected to further bioinformatic and statistical analyses, as described below.

#### Missing data

Clinicopathological characteristics of the patients comprised several missing values. Therefore, statistics reported in the Table 1, assessing prognostic properties of the individual characteristics, were calculated across the subset of patients without missing values. Number of patients used to calculate these statistics is indicated in the table (N). As there were no missing signature genes expression values, no data imputation was applied.

#### Statistical analysis

Gene expression data were normalized and background corrected, using R package NanoStringNorm v1.1.21^19^. Expressions were then normalized for technical assay variation using positive controls and background-corrected using negative controls. Subsequently, data were normalized for sample/RNA content variation by applying the housekeeping genes normalization method (housekeeping.geo.mean), as described in NanoString nCounter^®^ Expression Data Analysis Guide. Two housekeeping genes, *GAPDH* and *RPS18* were used as internal controls, as these have shown stable expression levels in our previous studies using normal and tumor tissues from head and neck cancer patients^9,18,20,21^. Normalized expressions levels were log-transformed and subsequently converted to Z-scores. Data were submitted to Gene Expression Omnibus (GEO), and are publicly available under GEO series number GSE108712.

Proportional risks were calculated as a linear combination of the Z-score-transformed expressions of *MMP1*, *COL4A1*, *P4HA2* and *THBS2*, applying original signature coefficients (0.25, 0.63, 0.45 and 0.34, respectively) as described by Reis *et al*.^9^. Patients were then stratified into two groups according to their proportional risks using the chosen threshold (see Results section). Hazard ratio between the two patient groups and associated p-values were then calculated using the *survdiff* function from the R package survival (version 2.38.3)^22^. Kaplan-Meier plots were generated using function survfit from the same package. Receiver operating characteristics (ROC) curve, area under ROC curve (AUC), and prediction accuracy were obtained using functions provide by the R package AUC (version 0.3.0)^23^.

#### Risk groups

When assessing the signature, patients were stratified into two groups according to predicted proportional risks, first using its median as a threshold, then using the optimized threshold that was selected to maximize significance of the resulting hazard ratio.

#### Development vs. validation data

The current validation was conducted using data from a fully independent cohort of patients, with no overlap with the cohort used for the signature development, or its first previously reported validation study^9^. Differences between the clinical and histopathological characteristics of the development and validation cohorts are summarized in Table 1.

#### Identification of protein-protein interaction partners and comprehensive pathway enrichment analysis

Physical protein interaction (PPI) partners of the 4 signature genes were obtained using the Integrated Interaction Database v2016-10 (IID; http://ophid.utoronto.ca/iid)^24^. We considered only interactions that were experimentally validated or computationally predicted in head and neck tissue. Subsequently, we performed a comprehensive pathway enrichment analysis using Pathway Data Integration Portal v2.5.9.3 (pathDIP; http://ophid.utoronto.ca/pathDIP)^25^.

## Supplementary information

Supplementary Material.
