# Supplementary material for: A 4-gene signature from histologically normal surgical margins predicts local recurrence in patients with oral carcinoma: clinical validation
Source: Sci Rep. 2020 Feb 3;10:1713. doi: 10.1038/s41598-020-58688-y (PMC6997450; doi:10.1038/s41598-020-58688-y)
Supplement: Supplementary file 1 — Supplementary Material. [file 41598_2020_58688_MOESM1_ESM.docx]

**Manuscript Title:**

A 4-gene signature from histologically normal surgical margins predicts local recurrence in patients with oral carcinoma: clinical validation.

**Authors:**

Patricia P. Reis†*, Tomas Tokar†, Rashmi S. Goswami, Yali Xuan, Mahadeo Sukhai, Ana Laura Seneda, Luis E. S. Móz, Bayardo Perez-Ordonez, Colleen Simpson, David Goldstein, Dale Brown, Ralph Gilbert, Patrick Gullane, Jonathan Irish, Igor Jurisica*, Suzanne Kamel-Reid*

† equal contributors

* corresponding authors

**Supplementary Material - Supplementary Table 1 and Supplementary Figures are included below.**

**Supplementary Table 1. Summary of 18 selected studies investigating molecular alterations in surgical resection margins in OSCC. Studies are organized in chronological order.**

| Study | N° of patients | Samples used for analysis | Method | Targets investigated | Main findings/conclusions |
| --- | --- | --- | --- | --- | --- |
| Nathan *et al*. 2002 | 25 | 25 tumors  25 margins  Head and Neck sites:  Larynx (14), Oral Cavity (7), Oropharynx (4) | IHC | eIF4E  TP53  MMP9 | eIF4E protein expression was suggested as a significant predictor of recurrence in HNSCC. |
| Shaw *et al*. 2007 | 20 | 60 tumors  52 margins  Oral cavity sites: Tongue, Base of tongue, Buccal mucosa | Quantitative pyrosequencing methylation | *P16*  *CYGB* | Quantitative pyrosequencing methylation may be promising for genetic analysis of surgical margins of oral cavity tumors. |
| Bilde *et al*. 2009 | 16 | 16 OSCC  16 margins  Oral cavity sites: Tongue (8), Floor of mouth (6), Retromolar trigone (1), Buccal mucosa (1) | IHC | TP53, P16, CHK2, Laminin-5, Glycosylated Oncofetal Fibronectin | Histologically normal tissues adjacent to oral carcinoma may have over-expression of TP53 and TP16. It is not clear whether these changes represent early malignant alterations or a response to cellular stress. |
| Sinha *et al*. 2009 | 38 | 38 tumors  30 margins (3 positive)  Oral cavity site: Tongue (38) | Quantitative methylation-specific PCR | *P16* | *TP16* promoter hypermethylation may serve as a useful biomarker of local recurrence in tongue carcinoma. |
| Heah *et al*. 2011 | 26 | 26 tumors  26 margins, 5 normal buccal mucosa  Head and Neck sites: Tongue (15), Maxilla (5), Buccal mucosa (3), Buccal sulcus (1), Alveolus (1), Unspecified (1) | IHC  FISH | TP53 | IHC analysis of TP53 protein expression and FISH analysis of TP53 gene was suggested as a screening tool for microinvasion of oral cancer. |
| Supic *et al*. 2011 | 47 | 47 tumors  94 margins  Head and Neck site: OSCC | Multiplex nested methylation specific PCR | *P16*, *DAPK*, *RASSF1A*, *APC*, *WIF1*, *RUNX3*, *CDH1*, *MGMT*, *hMLH1* | *DAPK* promoter hypermethylation in surgical margins may be a useful biomarker of poor survival in OSCC. |
| de Carvalho *et al*. 2012 | 55 | 23 tumors  55 margins, 25 healthy oral mucosa  Head and Neck sites: Oral cavity (41), Larynx (8), Oropharynx (6) | QRT-PCR (TaqMan Gene Expression Assay) | *THLH*, *EPCAM*, *MMP9*, *LGALS1*, *MET* | *MMP9*, *EPCAM* and *PTHLH* are frequently overexpressed in histologically negative surgical margins of HNSCC. *MMP9* and *PTHLH* overexpression in histologically negative margins was correlated to local failure and second primary tumors. |
| Shaw *et al*. 2013 | 48 | 48 tumors  5 deep and 5 mucosal margins  Head and Neck site: OSCC | Quantitative methylation-specific PCR | *P16*, *CYGB*, *TMEFF2*, *CDH1* | The gene panel tested did not add prognostic information to histopathological reporting of surgical resection margins in oral cancer. |
| Farah *et al*. 2015 | 18 | 18 tumors  Paired margins marked by white light and narrow band imaging  Head and Neck site: OSCC | GeneChip Human Genome U133  Plus 2.0 Arrays (Affymetrix) | Global gene expression profiles | Tumor, white light, and narrow band imaging tissues were segregated based on global gene expression profiles. Margins defined by narrow band imaging will increase success rates of surgical resection. |
| Jelovac *et al*. 2016 | 50 | 25 tumors  Paired margins  Head and Neck sites: Buccal mucosa (5), Maxillary mucosa (4), Mandibular mucosa (2), Lip (14) | DNA copy number analysis by QPCR, PCR/SSCP, Sequencing | *c-ERBB2*, *c-MYC*  (DNA amplification analysis), *H-RAS* (codon 12/13 mutation analysis) | *c-ERBB2* amplification was correlated with poor survival and disease relapse in OSCC. |
| Strzelczyk *et al*. 2016 | 56 | 56 tumors  56 margins  Head and Neck sites: Maxilla, Mandible, Floor of mouth, Tongue, Cheek | QRT-PCR for gene expression analysis | *p16*  *APC*  *MGMT* | There was no statistically significant difference in gene expression in tumor compared to margin tissues. Further molecular analysis on surgical margins is required. |
| Wang *et al*. 2016 | 71 | 71 tumors  284 margins  Head and Neck site: OSCC | LOH  IHC | D9S1747, RPS6, D9S162, *TP53*, *P14*, *P15*, *P16* | Detection of LOH on *P16* and D9S1747 was the most predictive biomarker of local recurrence. |
| Lin *et al*. 2017 | 120 | 120 tumors  Paired non-cancerous mucosa  Head and Neck sites: Tongue (46), Buccal mucosa (45), Gum (11), Floor of Mouth (7), Soft palate (5), Lip (4), Retromolar trigone (2) | Multiplex PCR,  Capillary array electrophoresis | Microsatellite markers D9S1748, D3S1079, THRB, D3S1234, D3S1300, IFNA.PCR2, D2S206, D21S236, D21S1433, D21S11 | Microsatellite instability in tumor-free surgical margins increases the risk of local recurrence. |
| Liu *et al*. 2018 | 145 | 145 tumors  651 surgical margins  Head and Neck site: OSCC | LOH and MSI  PCR, automatic fragment analysis | Microsatellite markers  D9S1748, THRB, D3S1300, IFNA.PCR2, D2S206, D21S236 | Microsatellite instability in surgical margin was associated with a higher risk of local recurrence. Molecular assessment of surgical margins can help identify patients at risk of local recurrence. |
| Eljabo *et al*. 2018 | 40 | 40 tumors  Paired margins, buccal mucosa swab  Head and Neck sites: Floor of mouth (16), lip (11), oropharynx (8), mandibular mucosa (3), buccal mucosa (1), tongue (1) | DNA copy number analysis by QPCR, Methylation analysis | *HER2*, *c-MYC*, *D2R*, *P14*, *P16*, HPV | *HER2* amplification as well as the existence of three co-alterations in margins was correlated with shorter disease-specific survival. |
| Lazarevic *et al*. 2018 | 6 | Cell cultures established from OSCC (6) and margins (6) | Gene expression analysis by QRT-PCR, Flow cytometry, Immunocytometry, Sphere formation, MTT assay | Stem cell marker expression: CD133, Nanog, Sox2, CD44, Oct4 | Subpopulations of cells within margin tissues may have stemness properties. |
| Strzelczyk *et al*. 2018 A | 56 | 56 HNSCC  56 margins | QRT-PCR for gene expression analysis | *TIMP3*, *SFRP1*, *SFRP2*, *CDH1*, *RASSF1*, *RORA*, *DAPK1* | *SFRP1* expression was statistically significantly lower in tumors than surgical  Margins. DAPK1 was over-expressed in poorly differentiated tumors. To find biomarkers associated with cancer development and to provide insight into the earliest stages of cancer development, attention should also be focused on molecular analysis of the surgical margins. |
| Strzelczyk et al. 2018 B | 75 | 75 oral cavity tumors  75 surgical margins | Methylation-specific PCR | *p16*, *APC*, *MGMT*, *TIMP3*, *CDH1* | Epigenetic changes were identified in cancer and surrounding mucosa; appropriate cancer risk assessment based on epigenetic alterations in surgical margins may help diagnosis and treatment. |

HNSCC: Head and Neck Squamous Cell Carcinoma. OSCC: Oral Squamous Cell Carcinoma. IHC: Immunohistochemistry. QRT-PCR: quantitative reverse transcription PCR. SSCP: Single Strand Conformation Polymorphism. None of the studies reported data validation in independent samples.

**References to Supplementary Table 1, listed in order of appearance:**

Nathan CO, Amirghahri N, Rice C, Abreo FW, Shi R, Stucker FJ. Molecular Analysis of Surgical Margins in Head and Neck Squamous Cell Carcinoma Patients. *Laryngoscope*. 2002;**112:**2129–40.

Shaw RJ, Hall GL, Woolgar JA, Lowe D, Rogers SN, Field JK, et al. Quantitative methylation analysis of resection margins and lymph nodes in oral squamous cell carcinoma. *Br J Oral Maxillofac Surg*. 2007;**45**:617–22.

Bilde A, Von Buchwald C, Dabelsteen E, Therkildsen MH, Dabelsteen S. Molecular Markers in the Surgical Margin of Oral Carcinomas. *J. Oral Pathol. Med*. 2009;**38:**72–8.

Sinha P, Bahadur S, Thakar A, Matta A, Macha M, Ralhan R, Gupta SD. Significance of promoter hypermethylation of p16 gene for margin assessment in carcinoma tongue. Head Neck. 2009;**31**:1423–30.

Heah KG, Hassan MI, Huat SC. p53 Expression as a marker of microinvasion in oral squamous cell carcinoma. *Asian Pac J Cancer Prev*. 2011;**12**:1017–22.

Supic G, Kozomara R, Jovic N, Zeljic K, Magic Z. Prognostic significance of tumor-related genes hypermethylation detected in cancer-free surgical margins of oral squamous cell carcinomas. *Oral Oncol*. 2011;**47**:702–8.

de Carvalho AC, Kowalski LP, Campos AH, Soares FA, Carvalho AL, Vettore AL. Clinical significance of molecular alterations in histologically negative surgical margins of head and neck cancer patients. *Oral Oncol*. 2012;**48**:240–8.

Shaw RJ, Hobkirk AJ, Nikolaidis G, Woolgar JA, Triantafyllou A, Brown JS, et al. Molecular staging of surgical margins in oral squamous cell carcinoma using promoter methylation of p16(INK4A), cytoglobin, E-cadherin, and TMEFF2. *Ann Surg Oncol*. 2013;**20**:2796–802.

Farah CS, Dalley AJ, Nguyen P, Batstone M, Kordbacheh F, Perry-Keene J, Fielding D. Improved surgical margin definition by narrow band imaging for resection of oral squamous cell carcinoma: A prospective gene expression profiling study. Head Neck. 2016;**38**:832–9.

Jelovac DB, Tepavčević Z, Nikolić N, Ilić B, Eljabo N, Popović B, et al. The amplification of c-erb-B2 in cancer-free surgical margins is a predictor of poor outcome in oral squamous cell carcinoma. *Int J Oral Maxillofac Surg*. 2016;**45**:700–5.

Strzelczyk JK, Gołąbek K, Krakowczyk Ł, Owczarek AJ. Expression profiles of MGMT, p16, and APC genes in tumor and matching surgical margin from patients with oral squamous cell carcinoma. *Acta Biochim Pol*. 2016;**63**:505–9.

Wang X, Chen S, Chen X, Zhang C, Liang X. Tumor-related markers in histologically normal margins correlate with locally recurrent oral squamous cell carcinoma: a retrospective study. *J Oral Pathol Med*. 2016;**45**:83–8.

Lin JC, Wang CC, Jiang RS, Wang WY, Liu SA. Impact of microsatellite alteration in surgical margins on local recurrence in oral cavity cancer patients. *Eur Arch Otorhinolaryngol*. 2017;**274**:431–439.

Liu SA, Wang CC, Jiang RS, Wang WY, Lin JC. Genetic analysis of surgical margins in oral cavity cancer. *Br J Surg*. 2018;**105**:e142–e149.

Eljabo N, Nikolic N, Carkic J, Jelovac D, Lazarevic M, Tanic N, Milasin J. Genetic and epigenetic alterations in the tumour, tumour margins, and normal buccal mucosa of patients with oral cancer. *Int J Oral Maxillofac Surg*. 2018;**47**:976–982.

Lazarevic M, Milosevic M, Trisic D, Toljic B, Simonovic J, Nikolic N, et al. Putative cancer stem cells are present in surgical margins of oral squamous cell carcinoma. *J BUON*. 2018;**23**:1686–1692.

Strzelczyk JK, Krakowczyk Ł, Gołąbek K, Owczarek AJ. (A) Expression profiles of selected genes in tumors and matched surgical margins in oral cavity cancer: Do we have to pay attention to the molecular analysis of the surgical margins? Adv Clin Exp Med. 2018;**27**:833–840.

Strzelczyk JK, Krakowczyk Ł, Owczarek AJ. (B) Aberrant DNA methylation of the p16, APC, MGMT, TIMP3 and CDH1 gene promoters in tumours and the surgical margins of patients with oral cavity cancer. J Cancer. 2018;**9**:1896-1904.

**Supplementary Figures**

**Supplementary Figure 1:**

Heatmap depicting expression of the 4-gene signature across all histologically negative margin samples. The associated risk score and recurrence status of the sample is shown in the right panel.

**
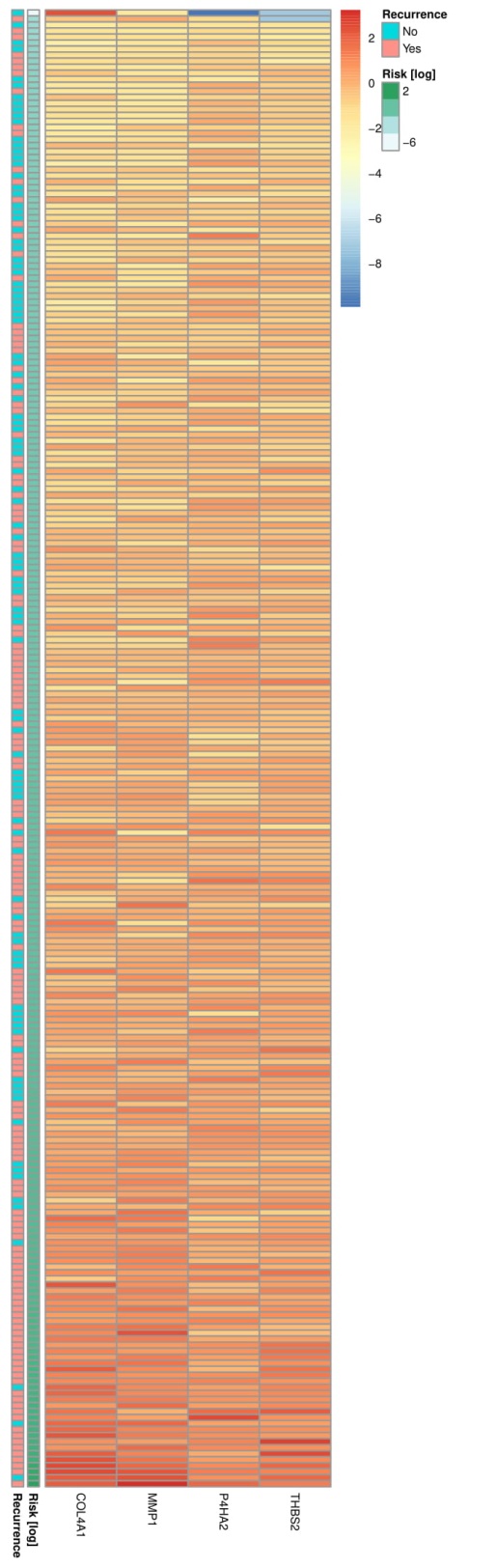
**

**Supplementary Figure 2:** Barplot depicting pathway enrichment analysis results. Height of the bar indicates statistical significance of the pathway enrichment; its colour indicates the pathway source database. Numbers above the bars indicate number of overlapping genes (overlap between the pathway genes and the four signature genes with their PPI partners).

**
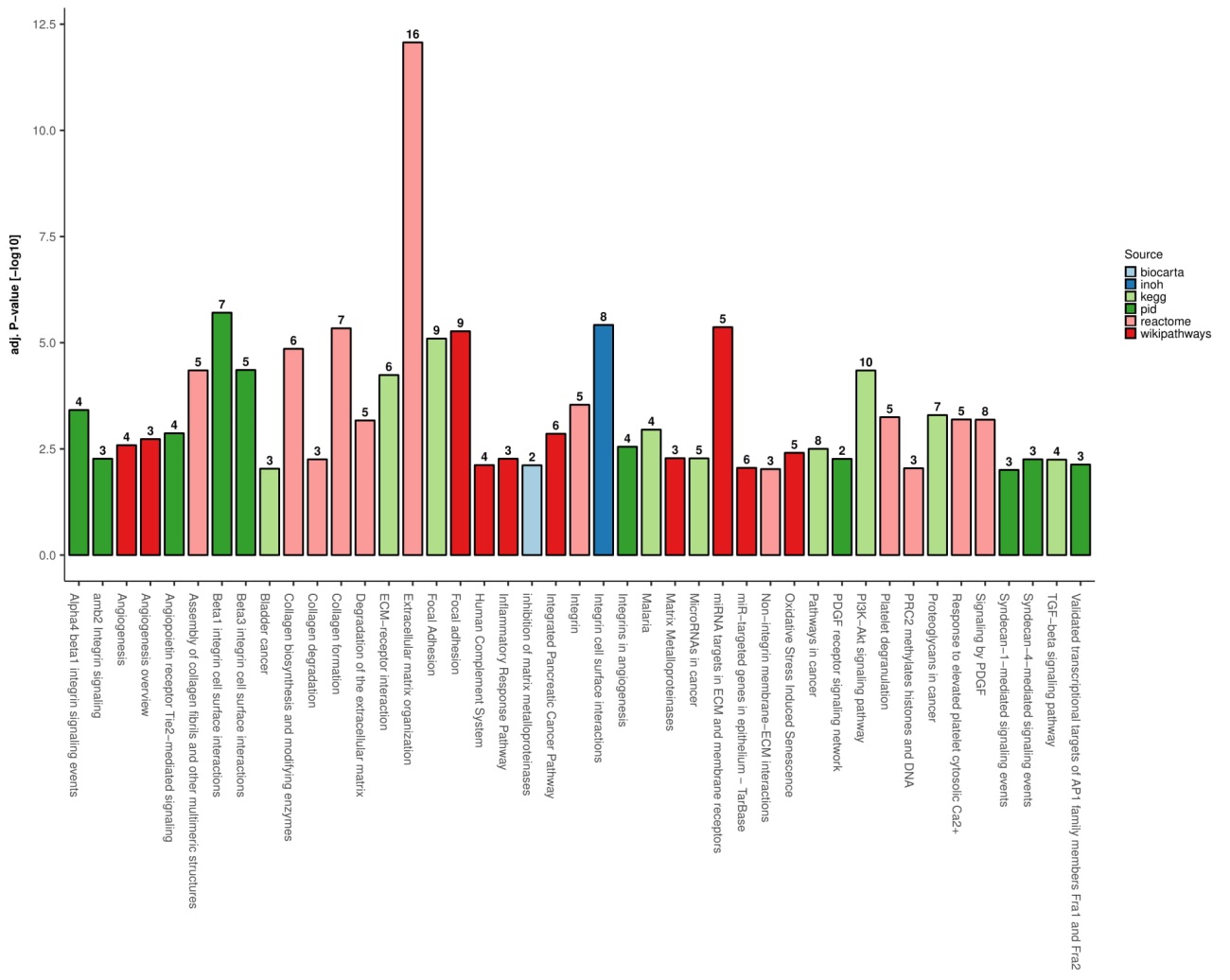
**
